# A natural compound from *Hydnophytum formicarium *induces apoptosis of MCF-7 cells via up-regulation of Bax

**DOI:** 10.1186/1475-2867-10-14

**Published:** 2010-05-04

**Authors:** Hasmah Abdullah, Azimahtol Hawariah Lope Pihie, Judit Hohmann, Joseph Molnár

**Affiliations:** 1 School of Health Sciences, Health Campus, Universiti Sains Malaysia 16150 Kota Bharu, Kelantan; 2 School of Bioscience and Biotechnology, Faculty of Science and Technology, Universiti Kebangsaan Malaysia, Bangi 46500, Selangor, Malaysia; 3 Institute of Pharmacognosy, University of Szeged, Hungary; 4 Institute of Medical Microbiology, University of Szeged, Hungary

## Abstract

**Background:**

*Hydnophytum formicarium *Jack is an epyphytic shrub that belongs to the family of Rubiaceae and is native to the tropical rain forests of the Asean region, which includes Malaysia. A flavanoid derivative, 7, 3', 5'-trihydroxyflavanone (3HFD), isolated from *H. formicarium *has been reported to have cytotoxic effects on the human breast carcinoma cell line MCF-7. The aim of the current study was to investigate the mode of cell death in MCF-7 cells treated with 3HFD. A DNA fragmentation assay was conducted on isolated genomic DNA, a TUNEL assay was used to determine the mode of cell death and Western blotting was used to evaluate the expression levels of Bax and Bcl-2. Immunofluorescence staining of MCF-7 cells was also performed to confirm the up-regulation of the Bax protein.

**Results:**

The ladder pattern resulting from the DNA fragmentation assay was a multimer of 180 kb. The morphological changes of cells undergoing apoptosis were visualised by a TUNEL assay over time. The percentage of apoptotic cells increased as early as 6 hours post treatment compared to untreated cells. Western blotting revealed up-regulation of the pro-apoptotic protein Bax. However, 3HFD did not affect expression of the anti-apoptotic protein Bcl-2.

**Conclusions:**

Our results provide evidence that plant-derived 3HFD was able to induce the apoptotic cell death of MCF-7 cells by increasing Bax expression level and makes 3HFD a promising agent for chemotherapy, which merits further study.

## Background

The tropical rain forests of Malaysia are known to be one of the most diverse forests for medicinal plants that may provide compounds for future anticancer therapies. It has been estimated that 250,000 species of flowering plants exist in the world. 150,000 of these species are found in Malaysian tropical rain forests [1]. Unfortunately, only 7.8% of these plants have been investigated for pharmacologically active compounds [2]. In the fight against cancer, novel chemotherapeutic agents are constantly being sought to complement existing drugs [2].

Cancer is one of the most serious, complex and diverse diseases. Modern treatments are effective at assaulting cancer cells, but these treatments may have unforeseen complications on neighbouring normal cells. The efficacy and safety of these treatments depends on the narrow therapeutic index that rates a drug based on its lethal dose and its therapeutic dose. The potential of plant remedies acting upon established malignancies is apparently limited. In 1955, screening of plant extracts for anti-cancer activity by the National Cancer Institute, United States demonstrated that less than four of every one thousand plant extracts tested contain compounds that demonstrate efficacy as anti-cancer agents. The efficacy and safety of these treatments depends on their ability to selectively target tumour cells [3].

However, epidemiological studies have revealed that eating a large amount of food from plant sources reduces the risk of cancer [4]. Therefore, identifying anti-cancer compounds in plant extracts has become a major effort recently. For example, Styrylpyrone Derivative (SPD) from the *Goniothalamus *sp. showed a profound anti-proliferative effect on a human breast cancer cell line (MCF-7) without cytotoxic effects on non-malignant cell lines (Chang Liver and MDBK) [5]. Plant extracts containing coumarins [6], flavanoids [7], acridone alkaloids [8] and diterpenes [9] have all been reported to have anti-cancer effects.

The plant botanically known as *Hydnophytum formicarium *Jack from the family Rubiaceae, which is native to Malaysia and Indonesia [10], has been investigated for anti-cancer properties [11]. Traditionally, this plant has been used to cure heart problems, treat chest pains and as an anti-inflammatory remedy [12]. A flavanoid derivative, 7, 3', 5'-trihydroxyflavanone (3HFD), was extracted from *H. formicarium *and was shown to have a potent anti-proliferative activity [11]. Although the number of studies on plant-derived anti-cancer agents is growing, the precise mechanism of plant-derived agents on the inhibition of cancer cell growth is not completely understood. Previous studies have reported that aberrant expression of the apoptosis-regulating genes from the Bcl-2 family contributes significantly to the pathogenesis of cancer. Hence, in this study, the mode of cell death induced by 3HFD treatment was evaluated in the human breast cancer cell line, MCF-7.

## Results

### DNA fragmentation analysis of MCF-7 cells treated with 3HFD

**Figure 1:**
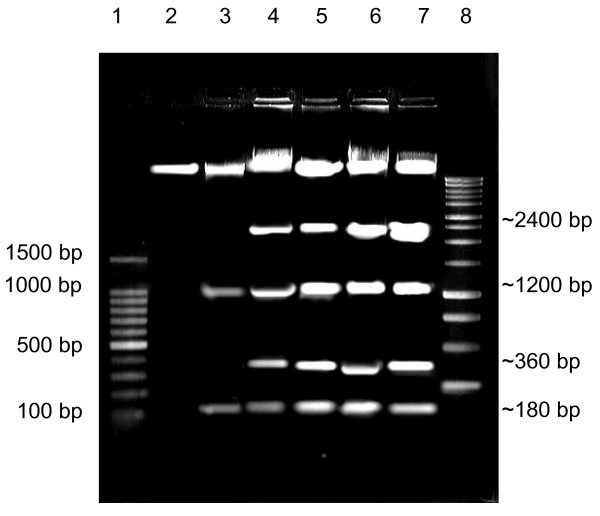
**DNA fragmentation analysis of MCF-7 cells treated with 3HFD**. Results of the DNA fragmentation analysis are presented as follows: untreated (lane 2); treated with 15 μg/ml of 3HFD for 72 h (lane 3); treated with 9 μg/ml of 3HFD for 12 h (lane 4), 24 h (lane 5), 48 h (lane 6) or 72 h (lane 7). Comparison with 100 bp (lane 1) and 1 kb (lane 8) molecular markers from Promega™ showed that lanes 4-7 were repeated fragments at 180 and 360 bp, whereas lane 3 exhibited a different fragmentation pattern with no bands corresponding to either 360 bp or 2400 bp were observed. This banding pattern may be explained by the detachment of the cells from the monolayer after 72 hours of incubation, because the cells lost the small fragment and the bigger fragment was not yet generated. The 180 bp fragments were weakly detected.

Reduction in MCF-7 cell viability [11] following treatment with 3HFD suggested the possibility of cell death. Figure 1 shows the treatment of MCF-7 cells with 3HFD based on the IC_50 _that was predetermined by an anti-proliferative assay [11]. According to Kerr and Harmon, DNA fragmentation is one of the hallmarks of apoptotic cell death that is induced by most anticancer agents [13]. Visualisation of DNA laddering, indicative of DNA fragmentation (Figure 1), was only significant when a large number of cells in a sample were engaged in the apoptotic death pathway; therefore, a different method of identifying these apoptotic cells was required. In the current study, we confirmed the presence of DNA fragmentation in cells treated with 3HFD by TUNEL (TdT-mediated dUTP Nick End Labelling) assay.

**Figure 2:**
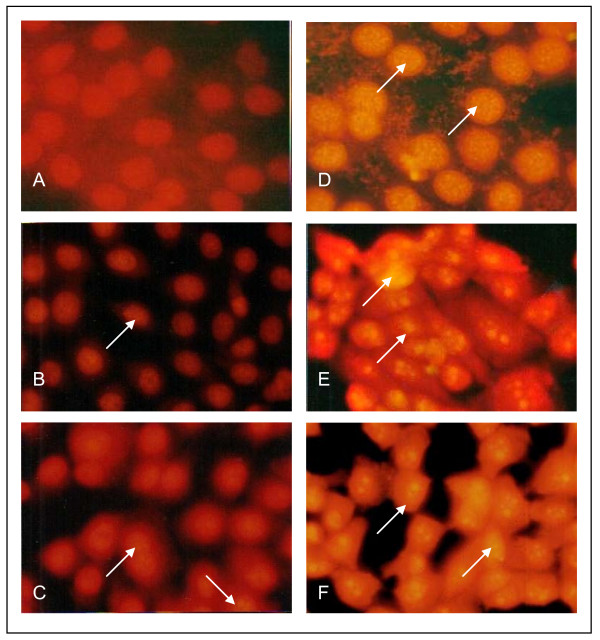
**TUNEL labelling of MCF-7 cells**. Untreated and 3HFD-treated cells were labelled using the TUNEL assay to detect DNA fragmentation. (A) In untreated cells, no fluorescence was detected in the nucleus, as the cells were not apoptotic and did not exhibit DNA fragmentation. (B) to (F) MCF-7 cells were treated with 9 μg/ml 3HDF for 6, 12, 24, 48 or 72 hours, respectively. Cells exhibit DNA fragmentation and nuclear condensation, characteristic of apoptosis. Arrows indicate representative apoptotic cells.

The TUNEL assay was developed as a method to identify individual cells that were undergoing apoptosis by labelling the ends of degraded DNA with the polymerase terminal deoxynucleotidyl transferase (TdT) [14], which catalyses the template-independent addition of deoxynucleotide triphosphates to the 3'-OH ends of DNA. In this study, 3HFD-treated cells were labelled using the TUNEL assay (Figure 2) to examine the morphology of the cells and to determine if DNA fragmentation occurs as a result of 3HFD. Generally, apoptotic cells exhibit small nuclei and have condensed chromatin. Eventually, the nuclear membrane disappears and membrane blebbing produces apoptotic bodies that contain cellular organelles and chromatin, as observed at 24 to 72 hours post 3HFD treatment (Figure 2D-F).

**Figure 3:**
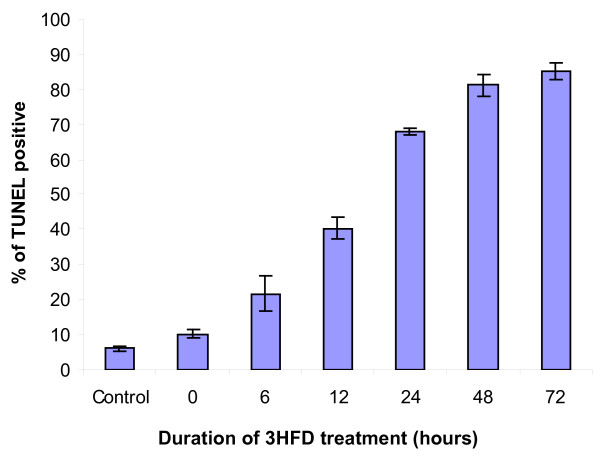
**Apoptosis levels as determined by TUNEL assay**. 3HFD treatment (9 μg/ml) significantly increased the level of apoptosis in MCF-7 cells as compared to untreated controls. Cells were observed up to 72 h after 3HFD treatment. Results are presented as the mean ± SEM of 3 independent experiments.

The percentage of apoptotic cells was determined by direct visualisation by fluorescence microscopy in 3 independent experiments (Figure 3). 3HFD treatment resulted in a 20% increase in the number of apoptotic cells after 6 hours compared to untreated cells.

### Expression of pro- and anti-apoptotic proteins in MCF-7 cells treated with 3HFD

**Figure 4:**
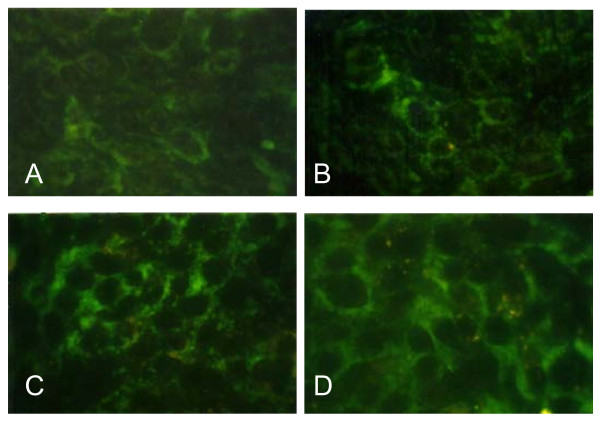
**Immunofluorescence staining of Bax protein in MCF-7 cells treated with 9 μg/ml 3HFD**. (A) Untreated MCF-7 cells at 0 h of 3HFD treatment showed a low level of Bax protein. (B), (C) and (D) MCF-7 cells after 6, 12 or 24 h of 3HFD treatment exhibited a marked increase of immunofluorescence for Bax protein.

**Figure 5:**
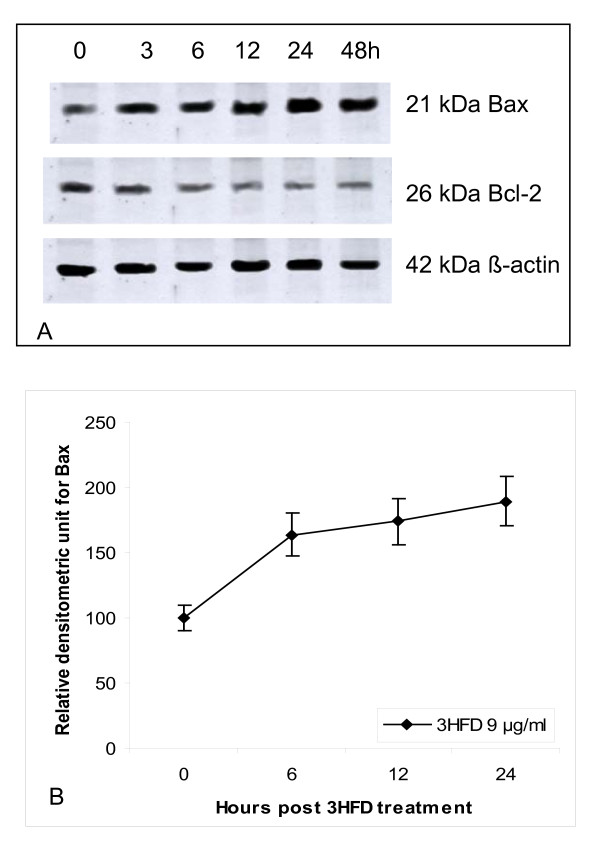
**Western blot analysis of Bax protein in MCF-7 cells treated with 9 μg/ml 3HFD**. (A) MCF-7 cells treated with 9 μg/ml 3HFD for the indicated times were resolved on a 15% PAGE and subjected to Western blotting. Bax protein expression increased as early as 3 h post treatment, whereas Bcl-2 levels were not altered and remained low throughout the experiment. (B)The intensity of Bax protein expression increased in a time-dependent manner. Results are presented as the mean ± SEM of 3 independent experiments.

The expression of the pro-apoptotic protein Bax is an early event that sensitises the cell to undergo apoptosis. Some models suggest that Bax up-regulation alone can commit a cell to apoptosis [15]. Cells treated with 3HFD exhibited a marked increase in fluorescence intensity (Figure 4A-D) when stained with a specific Bax antibody compared to controls. This result suggests that Bax expression is up-regulated (Figure 5A-B) in cells following treatment with 9 μg/ml 3HFD [11]. However, the anti-apoptotic, Bcl-2 levels were very low throughout the treatment period. Fragmentation of DNA, increasing levels of apoptosis and up-regulation of the pro-apoptotic Bax protein suggests a Bax-dependent apoptotic mechanism induced by 3HFD.

## Discussion

The need to develop more effective and less toxic anti-cancer drugs has prompted researchers to explore new sources of pharmacologically active compounds. This necessity is particularly important for more widespread types of cancers, such as lung, colon and breast cancers. Presently, chemotherapy and hormone compounds are not completely effective due to the non-specific mechanisms of action, non-specific and the presence of resistant cancer cells [16].

A natural product provides novel structural specialities that may qualify for new anti-cancer drugs. 3HFD, a compound isolated from *H. formicarium*, has cytotoxic effects against breast cancer cells similar to tamoxifen [11] without affecting normal cell lines, such as Chang Liver, Vero and MDBK. In this study, 3HFD was further investigated for the mode of cell death that reduced cancer cell viability. The data obtained from this study revealed that 3HFD induced apoptotic cell death.

The first morphological changes of apoptosis found in most cell types are contraction in cell volume and condensation of the nucleus, which allows the intracellular organelles, such as mitochondria, to retain their normal morphology. This change is followed by plasma membrane blebbing and nuclear fragmentation to form apoptotic bodies [17]. A closer look at the pattern of TUNEL staining in 3HFD treated MCF-7 cells suggests that DNA fragmentation is initiated at the nuclear periphery as described by Gavrielli et al. [14] and progresses towards the centre (Figure 1A-F) as observed in Kataoka and Tsuruo [17]. While TUNEL enables a determination of the fraction of cells undergoing apoptosis, DNA forms a characteristic laddering pattern on agarose gel electrophoresis that represents the biochemical changes involved in the fragmentation of chromosomes into nucleosome units [14]. As shown in Figure 1, multiple units of apoptotic DNA laddering were detected in 3HFD-treated MCF-7 that increased with the duration of treatment. This result was in agreement with the results obtained in the TUNEL assays and is illustrated by the increasing apoptotic scores (Figure 3) from approximately 20% at 6 hours to approximately 83% at 72 hours. These findings suggest that fully degraded nuclei are cleared from the cell. After a certain point, the change in gel electrophoresis pattern reflects only the ongoing intracellular activity of the putative endonucleases [18].

In this study, oligonucleosomal DNA laddering was observed. However, the laddering pattern shown by MCF-7 cells treated with 3HFD did not produced high molecular weight DNA fragments. Factors affecting lysosomal degradation are dependent on cell type and tissue [13]. This result was reported by Grem et al. [18], who studied MCF-7 and HL 60 cells treated with Pyrazoloacridine (PZA). When MCF-7 cells were treated with PZA, the laddering pattern was similar to the pattern observed in this study. In contrast, DNA fragmentation in HL-60 cells treated with PZA exhibited classic oligonucleosomal laddering as early as 6 hours after treatment with 10 and 25 μM PZA [18]. The production of megabase-sized DNA fragments is reported to be associated with the detachment of cells from the monolayer and decreased cell volume that does not disturb membrane integrity [19]. This might explains the current situation when, after 72 hours at a 2-fold higher concentration of 3HFD treatment, more than 80% of cells were TUNEL-positive, showing rapid DNA fragmentation, but with a lower percentage of cells detached from the substrate. Treatment of epithelial cancer cell lines with a specific DNA-damaging agent will produce high molecular weight DNA fragmentation in the absence of nucleosomal laddering [20-22]. Within the current study, the status of 3HFD as a DNA-damaging agent is unclear. To confirm this, a DNA synthesis study is needed to detect the relationship between inductions of parental DNA double stranded in concert with single-strand breaks [19].

Other than therapeutic agents that induce apoptosis, molecules that have been strongly implicated as major players in apoptosis are the *bcl-2 *oncogene [23] and Bcl-2 family proteins. The *bax-α *mRNA encodes the Bax protein, which promotes apoptosis due to its ability to form heterodimers with *bcl-2 *[24]. It has been reported that the *bcl-2 *gene is expressed in breast cancer cells [25], and the level of expression varies with alteration of some apoptotic stimuli [26]. Loss of *bcl-2 *gene expression has been linked to poor patient prognosis. However, it has not yet been determined whether *bcl-2 *or *bcl-2 *related genes play any role in the development of breast cancer [25]. Thus, in this study we have investigated the expression of Bax and Bcl-2 protein in MCF-7 cells treated with 3HFD.

In many human cancers, the anti-apoptotic Bcl-2 proteins are over expressed, or the pro-apoptotic proteins, like Bax, have reduced expression [27]. This results in resistance to a wide variety of cell death stimuli including chemotherapeutic drugs [28]. Bcl-2 protects against diverse cytotoxic insults, including γ and UV-irradiation, cytokine withdrawal, dexamethasone, staurosporine and cytotoxic drugs, while pro-apoptotic family members like Bax may act as tumour suppressors [29]. Therefore, finding new cytotoxic agents that are able to increase Bax expression or restore the ability of tumour cells to undergo apoptosis are vital. Our data demonstrate that 3HFD treatment down-regulated Bcl-2 and significantly up-regulated the expression of Bax in MCF-7 cells. Before 3HFD treatment, a low level of Bax was expressed in MCF-7 cells, as observed in Strobel et al. [30]. They reported that over expression of Bax in MCF-7 cells resulted in increased sensitivity to apoptosis.

Although the mechanism for activating the expression and function of Bcl-2, Bcl-X_L _and Bax is not fully understood, it is possible that the p53 molecule plays a role in this process [30]. This was demonstrated by the ability of wild-type p53 to down-regulate Bcl-2 and up-regulate Bax and proceed to programmed cell death [31-33]. The p53 status is dependent on the anticancer agent and type of cell line used. For example, SPD treatment bypasses the p53 mediated pathway in the ovarian cancer cell line Caov-3 [33]. This finding suggests that p53 might play a role in the regulation of apoptosis by SPD rather than through an elevation in p53 levels. In breast cancer cells, activity of p53 may initiate apoptosis without transcription [32].

3HFD treatment inhibits MCF-7 cell proliferation by inducing apoptotic cell death. The up-regulation of Bax protein expression suggests that 3HFD might be a potential anti-cancer agent in breast carcinoma. Additionally, 3HFD is part of a flavanoid group that may have estrogenic potency especially for the human estrogen receptor (ER) type Erβ as reported by George et al. [34]. This flavanoid group is thought to play a beneficial role in preventing breast cancer by competing with estrogens for binding to estrogens receptor (ER) [35].

## Conclusions

Our study demonstrates that 3HFD induced apoptosis in MCF-7 cells. Apoptosis was caused by decreasing the level of the anti-apoptotic protein Bcl-2 and up-regulation of pro-apoptotic Bax. This compound was also selective for MCF-7 cells because no effect was observed in non-malignant cell lines.

## Methods

### Cell culture

The cancer cell line MCF-7, and the non-cancerous cell lines MDBK, Chang Livers and Vero, were obtained from American Type Culture Collection (ATCC). MCF-7, MDBK and Chang Liver cells were maintained in DMEM. Vero cells were maintained in RPMI. DMEM and RPMI were supplemented with 5% foetal calf serum (FCS), penicillin-streptomycin and fungizone GIBCO™, Invitrogen.

### DNA fragmentation assay

The isolation of genomic DNA from treated cells was done as described by the manufacturer's protocol using DNAzol^® ^(Molecular Research Center Inc, USA). The isolated DNA was analysed on a 1.5% agarose gel, stained with ethidium bromide and viewed under Alpha Imager Image Viewer. The agarose gel was photographed and analysed.

### Apoptotic index

The morphological changes and apoptotic index of treated cells were analysed by TdT-mediated dUTP nick labelling (TUNEL) with the Apoptosis Detection Kit, Fluorescein (Promega) according to the manufacturer's protocol. To calculate the percentage of TUNEL positive cells, we counted cells from four random microscopic fields at 100× and 400× as described in [5].

### Immunofluorescence staining of Bax

Treated and untreated cells were fixed on slides and permeabilised with 0.2% Triton-X 100 for 20 min at 4°C. Then, slides were blocked with 2% foetal calf serum in PBS for 2 h at 37°C. After washing, cells were incubated overnight with monoclonal anti-Bax antibodies (Pharmingen) at a 1:200 dilution at 4°C. Next the slides were incubated with FITC conjugated secondary antibodies. Following washes, the slides were visualised with a fluorescence microscope.

### Western blotting

Protocols were slightly modified from [5]. Protein aliquots of 20 μg from both treated and untreated cells were separated on 15% SDS-polyacrylamide gels. The separated proteins were transferred onto polyvinyl-difluoride membranes (PolysScreen, Nen Life Science). The membranes were dried, preblocked in 5% non-fat milk in phosphate-buffered saline and 0.1% Tween-20 and incubated with primary antibody for Bax or Bcl-2 (Pharmingen) at a 1:1500 dilution. This was followed by incubation with horseradish peroxidase-labelled secondary antibodies to mouse IgG and detection on a Kodak BIOMAT x-ray film. Densitometry analysis was performed with a GS 670 Imaging Densitometer with the Molecular Analyst Software (BioRad, Hercules, USA). The membranes were reprobed with β-actin (Sigma) antibodies as an internal control

## List of abbreviations

ATCC: American Type Cell Culture Collection; Bax: Bcl-2-associated × protein; Bcl-2: B-cell lymphoma-2; Ca^2+^: calcium ion; Chang liver cells, normal liver cells; CO_2_: carbon dioxide; DMEM: Dulbecco's modified Eagle's medium; DMSO: dimethylsulfoxide; DNA: deoxyribonucleic acid; dUTP: deoxyuridine triphosphate; ELISA: Enzyme Linked Immuno Sorbent Assay; FBS: foetal bovine serum; HCl: hydrochloride acid; IC_50_: inhibition concentration to kill 50% of cells population; IgG: Immunoglobulin-G; MDBK cells: Madin Darby Bovine Kidney cells; PBS: phosphate-buffered saline; PVDF: polyvinyl-difluoride; SDS: sodium dodecyl sulphate; SSC: sodium chloride-sodium citrate; TdT: Terminal Deoxynucleotidyl Transferase; TUNEL: TdT-mediated dUTP nick-end labelling; h: hour; g: gram; bp: base pair.

## Competing interests

The plant compound, 3HFD, used in this study was the same as previously reported by the same group of authors [11]. However, the current study concentrated on the apoptotic mode of action towards cancer cells, whereas the previous study was on the chemical elucidation and anti-proliferative activity of the active compound from the same plant. Furthermore, the research was conducted 3 years ago.

## Authors' contributions

HA carried out the research and drafted the manuscript. JH, AHLP and JM participated in the design and coordination of the study. All authors read and approved the final manuscript.
